# Supplementary material for: Distribution of refractive errors and horizontal concomitant strabismus in Iraqi children
Source: PLoS One. 2026 May 27;21(5):e0349697. doi: 10.1371/journal.pone.0349697 (PMC13215519; doi:10.1371/journal.pone.0349697)
Supplement: Attachment — Submitted filename: Response_to_Reviewers 2.docx [file pone.0349697.s003.docx]

**Response to Reviewer#2
Manuscript ID: PONE-D-25-63958**
*Title: Distribution of refractive errors and horizontal concomitant strabismus in Iraqi children*

Dear Reviewer 2,

Thank you for the careful and constructive evaluation of our manuscript. We have revised the manuscript extensively in response to all comments. .

**Reviewer #2 – Comment 1**

**Comment:** *Use “assessed” instead of “accessed”.*

**Response:** We thank the reviewer for this correction. The erroneous wording has been removed during revision, and the relevant sentence has been edited accordingly in the revised manuscript.

**Reviewer #2 – Comment 2**

**Comment:** *The clinic-based design statement should be integrated more smoothly.*

**Response:** We thank the reviewer for this helpful comment. The study-design wording in the Methods section has been revised for smoother integration and clearer flow, while retaining the clarification that the findings reflect a clinic-based case-comparison sample rather than population prevalence.

**Reviewer #2 – Comment 3**

**Comment:** *The clinic-based design might have influenced oversampling of strabismus cases and should be addressed in the limitations.*

**Response:** We thank the reviewer for this important comment. We have now addressed this point explicitly in the Limitations section by clarifying that recruitment from referral pediatric ophthalmology centers may have overrepresented children with strabismus and clinically significant refractive abnormalities relative to the general pediatric population; accordingly, the observed distributions should be interpreted as clinic-based associations rather than population prevalence estimates..

We believe these revisions have improved the manuscript substantially and we appreciate the reviewers’ thoughtful guidance.

Sincerely,
Hassan A. Aljaberi (on behalf of all authors)
